# Identification of Stopping Points in GPS Trajectories by Two-Step Clustering Based on DPCC with Temporal and Entropy Constraints

**DOI:** 10.3390/s23073749

**Published:** 2023-04-05

**Authors:** Kang Wang, Liwei Pang, Xiaoli Li

**Affiliations:** 1 Faculty of Information Technology, Beijing University of Technology, Beijing 100124, China; plw@emails.bjut.edu.cn (L.P.); lixiaolibjut@bjut.edu.cn (X.L.); 2 Beijing Key Laboratory of Computational Intelligence and Intelligent System, Beijing 100124, China; 3 Engineering Research Center of Digital Community of Ministry of Education, Beijing 100124, China

**Keywords:** two-step clustering, density peaks clustering, temporal constraint, entropy constraint, stopping point extraction

## Abstract

The widespread adoption of intelligent devices has led to the generation of vast amounts of Global Positioning System (GPS) trajectory data. One of the significant challenges in this domain is to accurately identify stopping points from GPS trajectory data. Traditional clustering methods have proven ineffective in accurately identifying non-stopping points caused by trailing or round trips. To address this issue, this paper proposes a novel density peak clustering algorithm based on coherence distance, incorporating temporal and entropy constraints, referred to as the two-step DPCC-TE. The proposed algorithm introduces a coherence index to integrate spatial and temporal features, and imposes temporal and entropy constraints on the clusters to mitigate local density increase caused by slow-moving points and back-and-forth movements. Moreover, to address the issue of interactions between subclusters after one-step clustering, a two-step clustering algorithm is proposed based on the DPCC-TE algorithm. Experimental results demonstrate that the proposed two-step clustering algorithm outperforms the DBSCAN-TE and one-step DPCC-TE methods, and achieves an accuracy of 95.49% in identifying stopping points.

## 1. Introduction

The rapid progress of cell phones and automobiles has led to the flourishing of research based on GPS data. A key task in GPS data analysis is to identify user stopping points and uncover hidden semantic information to enhance user analysis and improve their experience. By analyzing user movement trajectories and stopping points, their interests and behavior habits can be determined, which can aid in business recommendations, pick-up point delineation, and even crime prevention.

One potential method for identifying stopping points along trajectories is to analyze their spatial–temporal characteristics, including velocity, duration, acceleration, orientation, and other relevant factors. Agamennoni et al. [1] and Yan et al. [2] employed a comparison of actual velocity with a predetermined threshold velocity at specific locations to identify stopping points, as well as low-speed or high-speed areas. Alvares et al. [3] mapped trajectories to possible activities by comparing them with a given threshold of stopping duration. While threshold-based methods can yield effective results in identifying stopping points in straightforward trajectories, the use of a single indicator or fixed threshold values is insufficient to achieve satisfactory clustering outcomes for GPS trajectories that record complex traffic situations with diverse travel modes.

Stopping points typically exhibit high-density characteristics, making density-based clustering algorithms the primary approach for their extraction [4,5,6]. However, trajectory points possess temporal characteristics, posing a significant challenge in incorporating temporal information into traditional Euclidean distance-based density clustering algorithms for stopping point extraction. To qualify the spatial–temporal density of data, Yang et al. [7] integrated the characteristics of neighborhood move ability, stay time, and evaluation factor noise tolerance. The method enabled the realistic extraction of various complex trajectories with high effectiveness. Furthermore, to evaluate the movement performance of different types of points, Yang et al. [8] developed a new moving index that combines point density and movement characteristics. A moving index Gaussian model was constructed based on this index to extract stopping points, which overcomes limitations of clustering based on stopping point features and reduces the pseudo-merging of stopping point clusters. However, this method struggles to differentiate transient stopover points from stopping points, and the analysis of moving point features at multiple scales makes the algorithm complex and inefficient for clustering.

Known for its ability to find arbitrarily shaped groups and isolate noise from other data, the DBSCAN (density-based spatial clustering of applications with noise) algorithm has been widely used in numerous fields, including automatic identification systems, anomaly detection, remote sensing, chemistry, and social sciences [9,10,11,12]. In recent times, researchers have employed DBSCAN and its various adaptations to extract stopping points from GPS trajectories in numerous studies [13,14,15,16,17]. Palma et al. [13] and Tran et al. [14] characterized the neighborhood as temporal linear and selected the core point in a cluster based on the minimum time criterion, rather than the minimum number of points, and selected along the trajectory distance to apply the improved DBSCAN algorithm. To prevent misidentifying stopping points due to increased local density caused by round trips, Gong et al. [15] proposed a two-step clustering algorithm C-DBSCAN. The first step involves extracting all stopping points while enforcing temporal and anomaly constraints. Specifically, the algorithm requires that all points in a cluster be temporally sequential and have an even distribution of direction change coefficient. In the second step, a support vector machine is utilized to distinguish between transient and active stopping points. Three features, namely stop duration, mean distance to the cluster centroid, and the shorter of the distances to home and to the workplace, are selected for supervised training. To enhance the performance of C-DBSCAN, Gong et al. [16] proposed an improved algorithm, DBSCAN-TE, which replaces the outlier detection process in the first clustering step with entropy constraints. The entropy index (EI), which measures the average amount of information and the degree of disorder within a system [17], is used to gauge the level of orderliness in the data. Specifically, the entropy constraint excludes clusters with an EI below a designated threshold, effectively filtering out slow-moving points with a regular pattern. However, the lack of an automated method for selecting optimal hyperparameters results in an uncertain level of accuracy for GPS data involving intricate traffic patterns and varying travel conditions. At the same time, the influence of input features of support vector machines on the correct classification rate has not been studied. The above drawbacks and the limitations of the dataset limit the scalability of the algorithm.

As another density-based clustering method, density-peak clustering (DPC) exhibits low complexity and high execution efficiency, while being capable of identifying arbitrarily shaped clusters without necessitating the use of multiple parameters [18,19,20]. Fu et al. [21] proposed a two-step algorithm to cluster stopping points from a single GPS trajectory. They customized three types of stopping points and clustered them using an improved density peak fast search and recognition algorithm. This method yielded better performance and accuracy than the DBSCAN method. Trajectory data often contain low-density areas that deviate from commonly visited locations, such as suburban tourism hotspots. In these situations, the DPC algorithm is better suited for analysis due to its introduction of a relative distance measure, which outperforms the DBSCAN method.

The methods discussed above, which are based on spatial–temporal characteristics and density clustering, have made significant progress in extracting stopping points from GPS trajectories. However, there are several drawbacks that need to be addressed. (1) Direct thresholds based on spatial–temporal characteristics are simple but cannot handle complex trajectories with various motion modes; (2) methods such as DBSCAN and its variations are computationally intensive and not feasible for addressing large volumes of data in the stopping point extraction process; (3) the challenges of mixed points resulting from slow-moving clusters and non-stopping points resulting from the trailing phenomenon have not received thorough investigation and effective solutions. To address these challenges, this paper proposes a novel two-step clustering approach based on DPC with coherence distance, as well as temporal and entropy constraints. This paper’s core contributions can be summarized as follows:

- An improved DPC method, DPCC, is proposed by introducing the coherence distance metric that considers both temporal information and the Euclidean distance during the DPCC clustering stage.
- Temporal and entropy constraints are introduced to address the mixing problem after clustering caused by temporal noncontinuous and slow-moving points.
- A two-step DPCC-TE clustering approach is proposed to resolve the interactions between subclusters that arises during single-step clustering, validating its efficiency compared with different methods.

The rest of this article is arranged as follows. Section 2 formulates the stopping point extraction problem and defines four types of points on trajectories, which will facilitate algorithm design. Section 3 introduces the improved DPC algorithm, which combines the temporal–spatial information using a coherence distance metric. Section 4 presents the procedures of the proposed two-step clustering method for extracting stopping points. Section 5 details the experiments conducted using Geolife GPS data, and Section 6 discusses the results. Finally, the conclusions are illustrated in Section 7.

## 2. Problem Description

In this paper, we refer to locations where individuals spend an extended duration of time engaged in activities such as work or shopping as “stopping points”. We assume the existence of a trajectory, denoted by $T=\{p_{1},\cdots ,p_{i},\cdots ,p_{n}\}$, where each trajectory point p={lat,lng,datetime,type} includes latitude (lat), longitude (lng), recording time (datetime), and point type (type). The objective of extracting stopping points is to identify the subset *Q* that consists of all stopping points in *T*, as defined by Equation (1).

(1) $$Q=\left\{p|p\in T,p.type=stop\right\}$$

**Figure 1:**
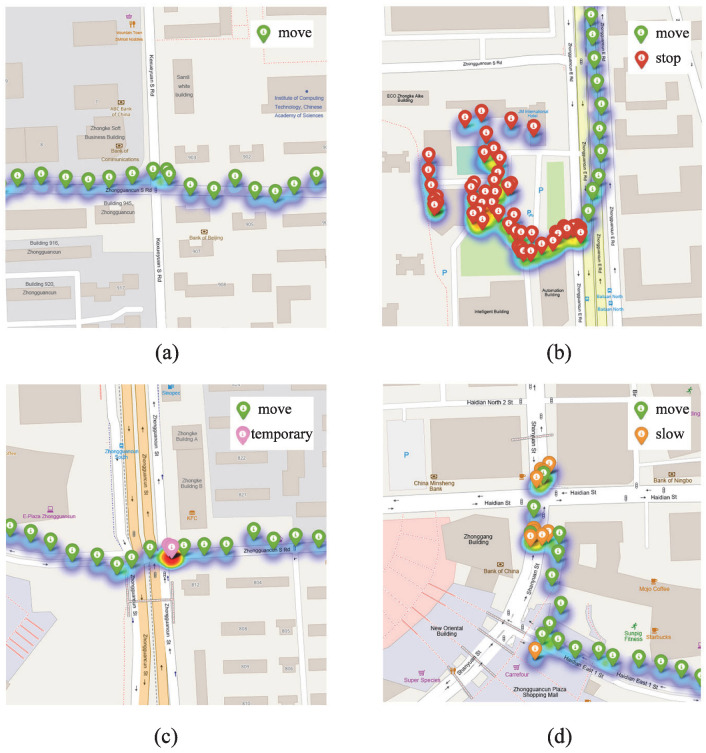
Typical distribution diagram of different types of points: (**a**) moving points, (**b**) stopping points, (**c**) temporary stopping points, (**d**) slow moving points.

This paper primarily focuses on the extraction of stopping points from GPS trajectories. To analyze the causes of misidentification in detail and facilitate algorithm design, non-stopping points are categorized into three distinct types: moving points, slow-moving points, and temporary stopping points. Using this categorization, all types of points in the trajectory can be denoted as p.type∈{move,slow,temporary,stop}. Figure 1 shows a typical distribution of the various types of points in a trajectory.

The following instructions outline how the figure should be interpreted:

- Moving points: the trajectory points generated by a user during the journey from one location to another, usually expressed as a straight line;
- Slow moving points: similar to the moving points, but with a lower velocity, resulting in an increase in local density;
- Temporary stopping points: the trajectory points generated when the user stops at a location for a short time, such as waiting for a cab, buying a bottle of water, etc.;
- Stopping points: the points where a person stays and engages in activities, such as shopping, gathering, working, etc. Unlike temporary stopping point, stopping points are purposeful and typically last for longer periods.

Based on above classification of trajectory points, reasonable speculations can be made about the characteristics of stopping points. Stopping points exhibit a relatively higher local density compared to other types of points. Additionally, the internal trajectories of clusters composed of stopping points exhibit a haphazard pattern. Given these inferences, it is possible to explore the potential interference of different point types in accurately identifying stopping points.

For moving points, passing through the same location at different times results in an increase in local point density in the region, potentially reaching the level of the stopping category. This indicates that relying solely on density as a criterion for classification is not practical.

For slow moving points, the local density increase is mainly due to a slower moving speed relative to the moving point. However, despite its higher density, the trajectory of slow moving points displays a greater degree of orderliness.

For temporary stopping points, the main difference with stopping points is that the user engages in simple activities such as waiting for a cab or purchasing water. This results in a smaller activity area and shorter dwell time compared to the stopping point.

Based on the above analysis, it can be seen that the extraction of stopping points is affected by various factors. In addition, the three types of non-stopping points in a cluster may also interact with the stopping points, leading to the interaction between sub-clusters, as shown in the example in Section 4.3. To address these challenges, we propose a two-step clustering method using improved density peak clustering for secondary clustering to extract stopping points.

## 3. Improved Density Peak Clustering Algorithm Utilizing Coherence Distance Metric

GPS tracking points typically include both latitude and longitude as well as time information. Traditional density peak clustering relies on the planar distance as the sole measure in the distance matrix, which is straightforward but does not take advantage of the time information available in GPS track points. As stopping points within the same area tend to have slower speeds and be closer to each other, this paper proposes an improved density-peak clustering algorithm that incorporates a coherence distance calculated using latitude, longitude, and time information rather than the traditional Euclidean distance. By doing so, the proposed approach enables a better utilization of temporal information for GPS tracking data clustering.

### 3.1. Raw Density Peak Clustering Algorithm

Proposed by Alex Rodriguez and Alessandro Laio in 2014 [18], DPC is a type of density-based clustering algorithm. In contrast to the traditional DBSCAN algorithm, the DPC algorithm relies on two primary assumptions:

- The cluster centers are surrounded by neighbors with lower local density.
- These cluster centers are at relatively large distances from any points with a higher local density.

Given the aforementioned assumptions, the density peak clustering algorithm requires the calculation of two metrics separately for each data point *i*: the local density $\rho_{i}$ and the relative distance $\delta_{i}$.

For the dataset $X=\{p_{1},p_{2},p_{3},\ldots ,p_{n}\}$, the distance between data points $p_{i}$ and $p_{j}$ is denoted by $d_{ij}$. The local density $\rho_{i}$ of data point $p_{i}$ is defined in two ways, truncated kernel and Gaussian kernel, or under the definition of truncated kernel, the local density $\rho_{i}$ is shown in Equation (2):

(2) $$\rho_{i}=\sum_{j=1}^{n}(\chi \left(d_{ij}-d_{c}\right)$$

where χ(x)=1 if x<0 and χ(x)=1 otherwise, and $d_{c}$ is a cutoff distance.

Under the definition of the Gaussian kernel, the local density $\rho_{i}$ is shown in Equation (3).

(3) $$\rho_{i}=\sum_{j=1}^{n}\exp\left(-\frac{d_{ij}}{d_{c}}\right)$$

The minimum distance $\delta_{i}$ from data point $p_{i}$ to other points with higher density is defined as Equation (4).

(4) $$\delta_{i}=\left\{\begin{array}{cc} \max_{j}\left(d_{ij}\right) & \rho_{i}=\max\left(\rho \right)\\ \min_{j:\rho_{j}>\rho_{i}}\left(d_{ij}\right) & \mathrm{others} \end{array}\right.$$

In the DPC algorithm, the appropriate local density is obtained by selecting the cutoff distance $d_{c}$. Once the local density $\rho_{i}$ and the relative distance $\delta_{i}$ of all data points are calculated, a two-dimensional decision map of data points is constructed based on $\rho_{i}$ and $\delta_{i}$. The clustering centers are then selected based on their distribution in the decision map. After identifying all the cluster centers, the remaining points are assigned to the cluster with the nearest point that has a higher density, completing the clustering process. Compared to the DBSCAN algorithm, the DPC algorithm can achieve better clustering results in remote low-density regions due to its consideration of the relative distance metric $\delta_{i}$.

### 3.2. DPC Using Coherence Distance Based on Spatio-Temporal Consistency

A GPS trajectory *T* that contains *n* trajectory points can be expressed as the sequence $\left\{p_{1},p_{2},\ldots ,p_{n}\right\}$. Each trajectory point $p_{i}$ records the longitude, latitude, altitude, time, and other information. For the purposes of this paper, only the latitude, longitude, and time information will be taken into account.

Density-based clustering methods, such as DBSCAN and DPC, rely on the distance between data points and clusters. However, when addressing GPS trajectories that include time stamps, direct density-based approaches run the risk of losing temporal information. To extract the user’s stopping points, which refers to a location where the user stays for a period of time and performs some activities, it is essential to combine the coordinates and timestamps. To enhance the utilization of temporal information in a trajectory and accurately extract a user’s stopping points, this paper introduces the concept of coherence distance, which is used to construct a new distance matrix referred to as the consistency matrix. The coherence distance is calculated as shown in Equation (5).

(5) $$coh\left(p_{i},p_{j}\right)=\arctan\left(\frac{d_{ij}}{\sigma }+\frac{d_{ij}}{t_{ij}}\right)$$

where $d_{ij}$ is the distance between trajectory points $p_{i}$ and $p_{j}$, σ is the deflation index, and $t_{ij}$ is the time difference between $p_{i}$ and $p_{j}$.

When the distance between trajectory points $p_{i}$ and $p_{j}$, denoted as $d_{ij}$, is shorter but the time difference $t_{ij}$ is longer, the coherence value $coh(p_{i},p_{j})$ is smaller. This indicates that the user has stayed in this area for a longer period of time, making it more likely that the two points belong to the same area. Thus, in calculating the local density $\rho_{i}$, we substitute the Euclidean distance in the distance matrix with the coherence distance. However, in computing the relative distance $\delta_{i}$, the Euclidean distance is used according to the second assumption of DPC. As a result, proposed DPCC (DPC with coherence distance metric) can incorporate both spatial and temporal information, instead of relying solely on physical distance.

## 4. Two-Step Clustering to Extract Stopping Points

**Figure 2:**
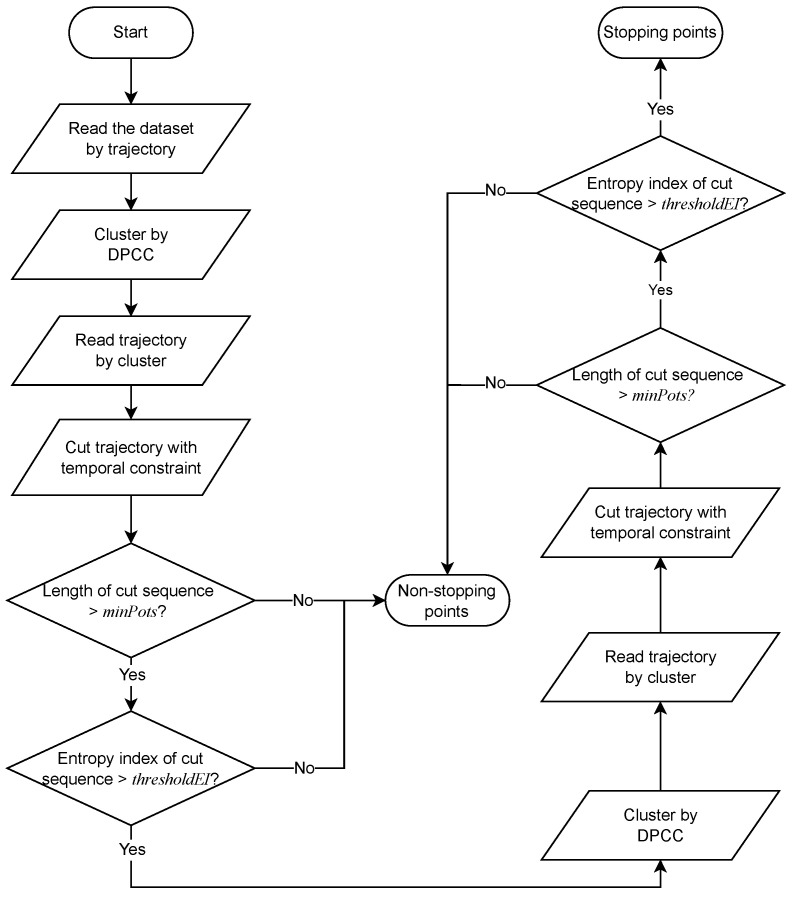
The structure of two-step DPCC-TE to extract stopping points.

Taking the characteristics of stopping points into account, this section employs density peak clustering with coherence distance based on temporal and entropy constraints and proposes a two-step clustering stopping point extraction method called DPCC-TE. First, trajectories are clustered using DPCC, each cluster is temporally segmented, and each trajectory sequence undergoes an entropy test, known as the first step DPCC-TE. Subsequently, each sequence is clustered, segmented, and entropy-tested again to eliminate some extended trajectories that are indistinguishable by the first step DPCC-TE. As a result, this whole algorithm is referred to as the two-step DPCC-TE. The specific workflow of this method is illustrated in Figure 2.

### 4.1. Temporal Sequence Constraint

After applying the improved density peak clustering algorithm introduced earlier, the clusters of trajectory are extracted, which are more aggregated both in time and space. However, even the temporal and spatial regions with high local density may still not necessarily be stopping points, as density-based clustering algorithms heavily rely on the accuracy of local density. As previously discussed in the problem description section, non-stopping points may exhibit an increase in local density due to round trips to the same location in different trajectories or within a single trajectory. To address this issue, this paper takes the following two approaches.

Multiple trajectories may pass through a particular location multiple times during human activities, such as an intersection used for commuting to work, resulting in higher local densities in that area. To mitigate local density increase, the trajectories in the dataset are processed individually rather than as a whole collection of trajectories. This approach effectively avoids the accumulation of density at the location of interest. However, a similar situation may occur in a single trajectory, where non-stopping points cannot be differentiated using the aforementioned method.

**Figure 3:**
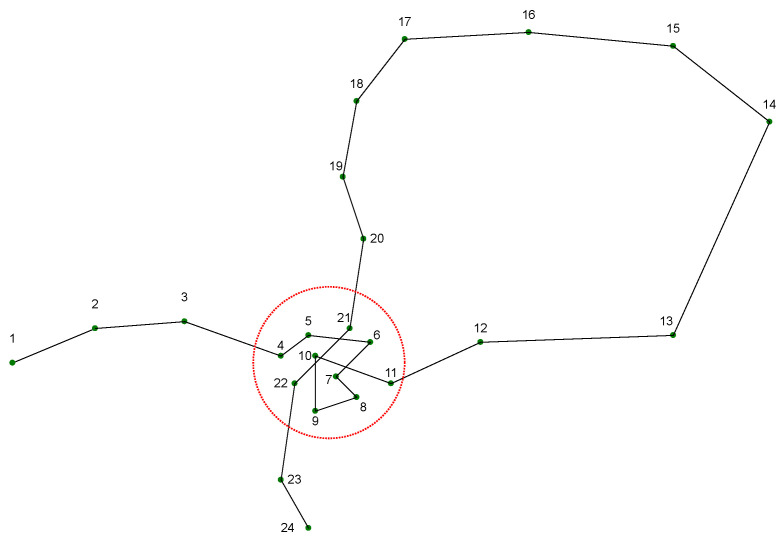
An example of temporal non-continuous points in cluster results.

As shown in Figure 3, the cluster contains stopping points 4–11 and moving points 21–22. Some of the moving points pass through this area, resulting in their clustering into stopping points.

However, it is observed that there exists a discontinuity in time between the moving and stopping points in the cluster. If the trajectory points are marked in chronological order, the trajectory points within the cluster exhibit a jumping index, as seen in Figure 3 at 11 and 21. Consequently, the points in the cluster can be divided into two parts, points 4–11 and points 21–22. Based on this, the temporal sequence constraint can be defined as follows.

Temporal sequence constraint: in a cluster, the trajectory sequence must demonstrate continuous growth over time without any discontinuities, with each coordinate point being indexed chronologically. Following the clustering process, the resultant cluster will be partitioned into sub-clusters that conform to a strict order of sequence increase, one by one.

### 4.2. Entropy Constraint

After applying clustering and temporal constraints, different trajectory sequences are obtained. Although these trajectory sequences are not connected to each other, the points within each sequence are continuous in time. However, due to the presence of slow moving points, a relatively low moving speed also leads to the increase in local density, making them be misidentified as stopping points. Nevertheless, it can be observed that although the slow moving points are dense, they move mainly in one direction, as indicated by the similarity of the direction angle between the front and rear trajectories. This reflects the orderliness of the trajectory sequence. On the contrary, the trajectories of the stopping points exhibit a disorder of directional angles due to interactions within a certain region. To quantify the disorder in the trajectory sequence, we use the entropy index, specifically, the Theil index, an economic indicator first proposed by Henri Theil to measure economic inequality [22], to measure the orderliness of the trajectory sequences.

The entropy constraint: Trajectory points exhibit directional regularity when a person just passes by, but they exhibit directional chaos when a person stays in one place. To quantify the regularity of the trajectories, this paper uses the entropy index to describe the chaos degree of trajectories. Specifically, for a trajectory sequence, the directions of the trajectory refer to the directions formed by the orientation angles between each pair of consecutive points. When the entire space of Cartesian coordinates is divided into *D* groups equally, the entropy index of trajectory sequence *S* is calculated by Equation (6),

(6) $$EI_{S}=-\sum_{d=1}^{D}\left(\frac{n_{d}}{M}ln\left(\frac{n_{d}}{M}\right)\right)$$

where *M* is the number of intervals for the direction and $n_{d}$ is the count of directions falling into interval *d* in the trajectory sequence *S*.

### 4.3. Two-Step Clustering Based on DPCC-TE

**Figure 4:**
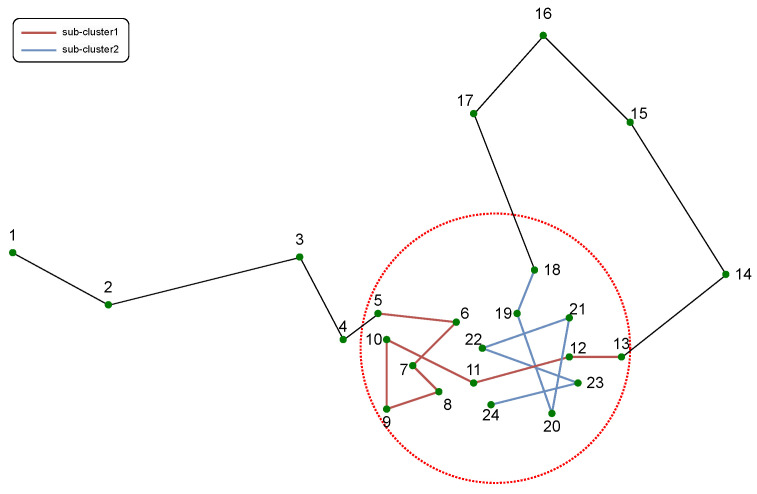
An example of interactions between sub-clusters in clustering results.

By employing methods with temporal and entropy constraints, we can remove those points in the trajectory that are erroneously classified as stopping points due to the local density increases caused by multiple round trips or slow GPS carrier movements. However, in reality, the clusters obtained through clustering may contain multiple types of blended points, leading to complications. For example, consider the scenario illustrated in Figure 4:

As shown in Figure 4, the points inside the red circles are extracted as a cluster in this trajectory. After applying the temporal sequence constraint, this cluster is divided into sub-cluster 1 (points 5–13) and sub-cluster 2 (points 18–24). It should be noted that in sub-cluster 1, the moving point trajectory (points 12–13) extends from the stopping points cluster (points 5–11), causing a trailing phenomenon. However, due to the excess of stopping points over moving points in sub-cluster 1, its entropy index is lower than that of a sequence composed entirely of stopping points, but still exceeds the threshold, resulting in sub-cluster 1 being identified as a stopping cluster. The occurrence of this phenomenon is due to the mutual influence of different sub-clusters, resulting in the presence of different types of points in the sub-cluster. Moreover, this phenomenon can occur in other combinations, making the extraction of stopping points more challenging.

The issue is addressed by proposing a two-step DPCC-TE clustering algorithm, which is shown as Algorithm 1. As previously mentioned, trajectories are first clustered, and then the resulting clusters are subjected to temporal and entropy constraints to derive the stopping points, which is called the first step. For the extracted clusters, we then repeat this process again to obtain the final stopping points. This algorithm is referred to as the two-step clustering algorithm in this paper, and its detailed procedure is shown in the next subsection. The proposed two-step clustering algorithm effectively addresses the issue of subcluster interaction, which is a common problem in density-based algorithms when addressing different points located in the same spatial location but recorded at different times. Following the first round of DPCC-TE, the algorithm divides clusters into temporally continuous subclusters. Subsequently, the local density of these subclusters is recalculated before the second-step clustering process. For subclusters that have tailing caused by moving points, the local density is recalculated using a new threshold to remove these points. However, other types of points require an additional round of DPCC-TE to eliminate them.
**Algorithm 1** Two-Step Clustering to Extract Stopping Points**Require:** Trajectory $T=\{p_{1},p_{2},\ldots ,p_{i},\ldots ,p_{n}\}$, where $p_{i}=\left\{lat_{i},lng_{i},datetime_{i}\right\}$.**Ensure:** $Q=\{p_{1},p_{2},\ldots ,p_{j},\ldots ,p_{m}\}$, $\forall p_{j}\in T$ and $p_{j}$ is stopping point.1:Read trajectories *T*.2:**for** i=1 to *n* **do**3:    **for** j=1 to *i* **do**4:        $coh\left[i,j\right],coh\left[j,i\right]=coh\left(p_{i},p_{j}\right)$5:        $dis\left[i,j\right],dis\left[j,i\right]=dis\left(p_{i},p_{j}\right)$6:    **end for**7:**end for**8:**for** i=1 to *n* **do**9:    Calculate coherence distance based local density $\rho_{i}$ by Equation (3), where $d_{ij}=coh\left[i,j\right]$10:    Calculate minimum distance $\delta_{i}$ by Equation (4) with Euclidean distance11:**end for**12:Obtain cluster centers by decision diagram13:Get one-step clusters $C_{i}$ with DPCC.14:**for** cluster in $C_{i}$ **do**15:    Get $subCluster_{i}^{k}$ by temporal constraint.16:**end for**17:**for** subCluster in $subCluster_{i}^{k}$ **do**18:    **if** EI(subCluster)≥entropyIndexThreshold **then**19:        $candidateCluster^{1}.append\left(subCluster\right)$20:    **end if**21:**end for**22:**for** cluster in $candidateCluster^{1}$ **do**23:    Obtain $candidateCluster^{2}$ using the method described in lines 2 to 24.24:**end for**25:Get the set of stopping points set $Q=\{p|p\in candidateCluster^{2}\}$

### 4.4. Algorithm Time Complexity Analysis

Let the number of trajectories in the dataset be *N* and the number of coordinate points of the *i*th trajectory be $n_{i}$; then, the computation of the *i*th trajectory is

Calculating the distance matrix, time complexity $O(n_{i}^{2})$;

Calculating the local density, time complexity $O(n_{i}^{2})$;

Selecting clustering centers, time complexity $O(n_{i}$);

Time order constraint and entropy constraint, time complexity $O(n_{i})$.

Therefore, for the *i*th trajectory, its time complexity is $O(n_{i}^{2})$. For the whole dataset, the time complexity depends on the trajectory with the highest number of trajectory points.

In contrast to the original DPC, this paper does not increase the time complexity when addressing a single trajectory. Moreover, for the whole dataset with *N* points totally, the algorithm’s complexity decreases from $O(N^{2})$ to $O\left(max{\left(n_{i}\right)}^{2}\right)$, and its worst case degenerates to $O(N^{2})$ due to the use of split trajectory operation. Compared to other clustering methods that use trajectory segmentation and have a time complexity of $O(max{\left(n_{i}\right)}^{2})$, this algorithm clusters twice, resulting in a constant increase in time consumption, but no increase in the computation complexity.

## 5. Results

### 5.1. Dataset Description

The study utilized the GeoLife GPS Trajectories dataset, which was gathered by Microsoft Research Asia in the Earthlife project [23]. The dataset contains GPS trajectories recorded by 182 users over a period of more than four years, from April 2007 to August 2012. The trajectories are represented by a series of time-stamped points that include latitude, longitude, and altitude information. The dataset comprises 17,621 tracks, with a total distance of 1,292,951 km and a total duration of 50,176 h. The trajectories were recorded using various GPS recorders and phones with different sampling rates, and 91.5% of them were recorded in a dense representation, such as every 1/5 s or every 5–10 m per point. Due to the absence of validation data, manual labeling was used to determine the type of trajectory points, with the judgment criteria outlined in the problem description section. In this experiment, the dwell time is calculated by subtracting the timestamps of the first arrival point and the last departure point in the area of the type.

**Figure 5:**
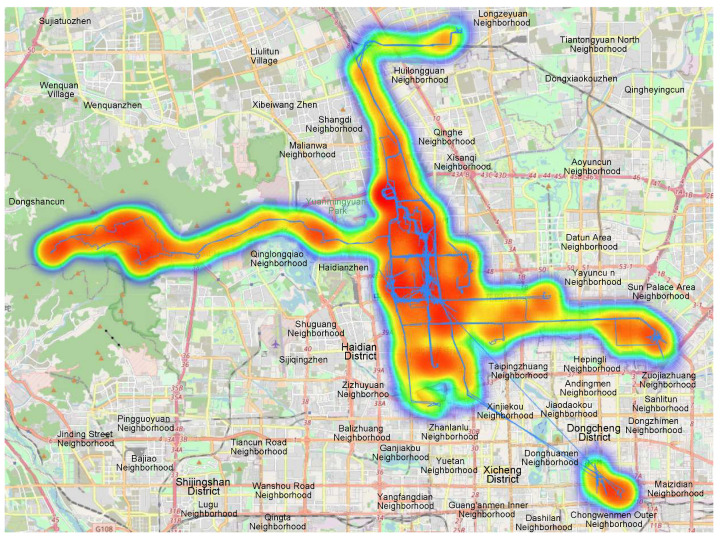
Track data distribution of user 001.

The manual annotation process covered all trajectories of user 001, which contained 54,322 trajectory points and 71 trajectories in total. The data were recorded in Beijing city between 23 October 2008 and 14 December 2008. The specific trajectory data can be found in Table 1. In the original dataset, field 3 was set to 0, and it should be noted that field 5 along with field 6 and 7 represented the same date/time in different formats. The distribution of all trajectories is depicted in Figure 5.

### 5.2. Validation of the Entropy Constraint

Section 4.2 describes the principle of the entropy constraint, which is used in the process of extracting stopping points from trajectory data. The entropy index is a measure of the degree of disorder in a trajectory sequence, with higher values indicating more chaotic behavior. The prerequisite for the validity of the entropy constraint in stopping point extraction is that the trajectory sequence formed by different types of trajectory points will produce different entropy indices. A crucial prerequisite for using the entropy constraint in stopping point extraction is that trajectory sequences composed of different types of points produce distinct entropy indices. Building on the trajectory classification system presented in Section 2 and the definition of the entropy index, it is expected that the degree of disorder for sequences composed of stopping points, temporary stopping points, slow moving points, and moving points will decrease in order, with their entropy gradually decreasing.

**Figure 6:**
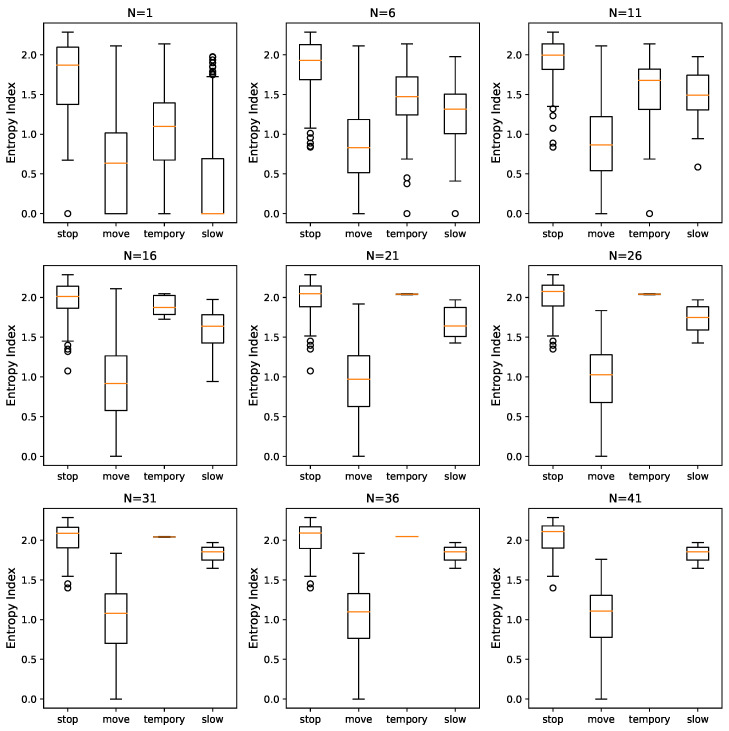
Box plot of entropy index for GPS pieces with different minimum lengths.

To verify the effectiveness of the entropy constraint, each trajectory sequence in the original dataset is cut into a series of continuous trajectory sequences of the same point type. Without imposing a length constraint, 3106 sequences are obtained from 71 trajectories. Then, the entropy indices of these trajectory sequences are calculated to obtain the box plot to visualize the results. At the same time, to avoid the influence of too-short trajectory sequences on the results, we varied the minimum lengths of trajectory sequences from 1 to 41 in steps of 5. The resulting final box plot is shown in Figure 6.

Figure 6 illustrates that the entropy index distribution of trajectory sequences consisting of stopping points, temporary stopping points, slow moving points, and moving points decreases in descending order when the minimum sequence length N≥6. However, for N=1, the distribution of slow moving points is similar to that of moving points due to the inclusion of short trajectory sequences. When N≥ 21, the entropy index of temporary points is contracted to a single value. Moreover, as the minimum sequence length increases, the numbers of temporary stopping points and slow moving points shrink to very narrow intervals, and the values of most stopping and moving points tend to stabilize at Q3 and Q1, respectively, while the number of outliers decreases.

### 5.3. Extraction Results of Different Algorithms for Extracting Stopping Points

**Figure 7:**
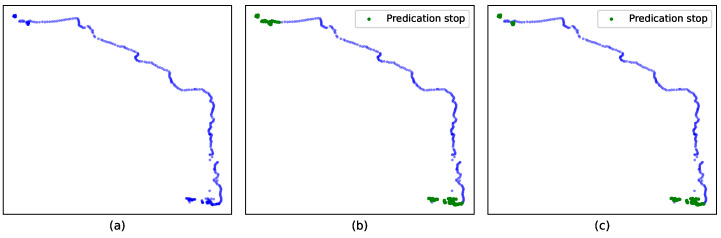
The result of stopping point extraction: (**a**) original trajectory, (**b**) one-step DPCC-TE, (**c**) two-step DPCC-TE.

Previous experiments have confirmed the effectiveness of the entropy constraint and indicated that the optimal threshold for the entropy index is approximately 1.5. To visually verify the effectiveness of two-step clustering, we performed stopping point extraction operations on track 6 using both one-step clustering and two-step clustering. The one-step clustering was carried out when the cutoff distance was set to $d_{c}=2\%$ in DPCC. The entropy threshold value was set to thresholdEI=1.5, the minimum number of points was minPots=12, and the number of intervals for direction was M=8. For the two-step clustering, we set the entropy threshold to thresholdEI=1.0, while retaining the other parameters identical to those used in one-step clustering. The results were obtained by applying the second step of the two-step clustering, and the visualization of the outcomes is presented in Figure 7.

Figure 7 demonstrates the effectiveness of two-step DPCC-TE in reducing misidentified stopping points in the upper left corner. In the case of 6, the accuracy of stopping point extraction increases from 96.14% with one-step DPCC-TE to 100% with two-step clustering.

**Figure 8:**
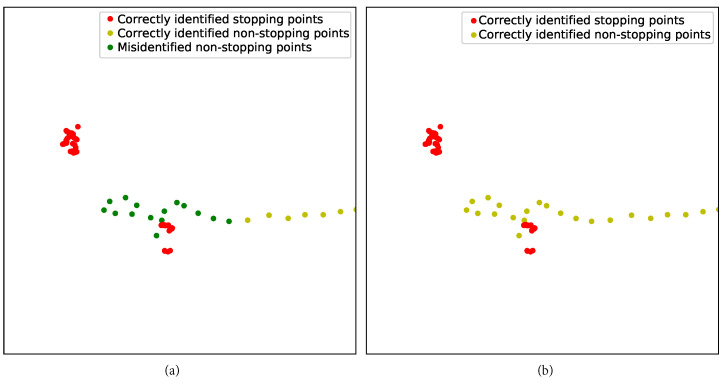
The local stopping point extraction results of the track 6 trajectory: (**a**) one-step DPCC-TE, (**b**) two-step DPCC-TE.

The region in the upper left corner of track 6 with a longitude range of 116.305–116.310 and a latitude range of 40.010–40.015 is analyzed separately. The results are presented visually in Figure 8.

Figure 8 demonstrates the effectiveness of two-step DPCC-TE over one-step DPCC-TE in accurately identifying stopping points. In one-step DPCC-TE, some green dots are present, indicating false identification as stopping points. However, in two-step DPCC-TE, all green dots have been eliminated. The second-step DPCC-TE successfully eliminates non-stopping points that could not be resolved by one-step DPCC-TE.

To evaluate the overall effectiveness of two-step DPCC-TE, DBSCAN-TE, one-step DPCC-TE, and two-step DPCC-TE are performed on 54,322 trajectory points and 71 trajectories separately. The results are shown in Table 2, which displays the indicators of different algorithms.

As shown in Table 2, for the extraction of stopping points, the two-step DPCC-TE achieves the highest accuracy of 93.63%, precision of 85.02%, and F1 score of 0.8822. At the same time, one-step clustering and DBSCAN-TE exhibited a decreasing trend in performance. Although DBSCAN and one-step clustering achieved the highest recall, too many non-stopping points were incorrectly identified, resulting in poor accuracy and precision. Meanwhile, as all methods were processed with a single trajectory at a time, DBSCAN-TE had the shortest processing time of 7 m 6.7 s, and the two-step clustering method had the longest processing time due to two rounds of clustering. The one-step clustering method computed two distance matrices and took slightly more time than DBSCAN-TE. These results are in accordance with the complexity analysis discussed previously in Section 4.4.

## 6. Discussion

The experimental results demonstrate the effectiveness of the proposed two-step clustering algorithm based on DPCC in identifying stopping points from trajectory data with timestamps. The second clustering step eliminates the mutual interference between different sub-clusters and improves the accuracy of stopping point extraction.

Distinct distribution characteristics are observed among various types of trajectories, with trajectories in the stopping point region typically displaying greater disorder in direction. Figure 6 illustrates that the entropy index of stopping points, temporary stopping points, slow moving points, and moving points decreases when N≥6. This result is consistent with both their physical meaning and the previously analyzed assumption of entropy index. For short trajectory sequences with N=1, there is a possibility of mixing with moving points. On the other hand, when N≥21, the number of temporary stopping points decreases significantly. These findings confirm the effectiveness of the entropy constraint in the proposed two-step clustering algorithm.

The proposed two-step DPCC-TE algorithm is density-based. However, the traditional calculation of density relies solely on spatial information, which causes the points in the same space area but at different times to have an impact on clustering. This is also the root cause of the situations illustrated in Figure 4 and Figure 8. To address this challenge, this paper introduces the temporal aspect using two methods: (1) incorporating the consistency distance and (2) utilizing time-series segmentation. These techniques aim to account for the impact of time on density calculation, thereby enabling more accurate and robust stopping point identification.

As depicted in Figure 8a, even after the initial DPCC-TE clustering step, there are still instances of misidentified stopping points due to increased local density of non-stopping points and mixed multiple-type clusters in the trajectory. To address this issue, a second DPCC-TE clustering step is performed to remove the extended trajectory pieces of the moving points in the sub-clusters, as shown in Figure 8b. This approach effectively eliminates the extended trajectory, leading to improved accuracy in stopping point recognition.

The performance comparison results in Table 2 indicate that the proposed two-step DPCC-TE algorithm outperforms the other two methods in terms of accuracy, precision, and F1 score, demonstrating its effectiveness in extracting stopping points from GPS trajectories. Although the processing time of the two-step clustering method is longer than the other two methods due to two rounds of clustering, its performance is significantly better, indicating that the increased processing time is worthwhile. Meanwhile, it should be noted that the algorithm has multiple parameters, including two rounds of clustering, cutting, and entropy constraint operations, making optimization challenging. To address this issue, future research can leverage prior knowledge from the first round of clustering to achieve adaptive parameter adjustment during the second round of clustering. Additionally, the output of the proposed algorithm can be utilized as input to other classifiers to further enhance stopping point recognition performance.

## 7. Conclusions

Stopping points extraction is a crucial task in the process of trajectories analysis. In this paper, we present a novel clustering algorithm called two-step DPCC-TE for extracting stopping points from GPS trajectories. The main focus is to develop an effective approach that combines both temporal and spatial distance information to extract stopping points accurately. First, a new consistency distance index is introduced to the design of density peak cluster, which effectively combines the temporal and spatial distance information. Next, the temporal sequence constraint is utilized to guarantee the temporal consistency of points within a cluster, while an entropy constraint ensures stopping points extraction by the chaos degree of trajectories. Finally, a second-step clustering is applied to each subcluster to solve the problem of subcluster mixing that cannot be eliminated by a single clustering step. Simulation and experimental results demonstrate the effectiveness of the method.

This experiment demonstrates that the entropy index of different types of trajectory points follows a certain order of magnitude, which does not vary with the length of the trajectory, and can be used to distinguish stopping points from non-stopping points. Meanwhile, compared with DBSCAN-TE and single-step clustering, the two-step clustering achieved best accuracy in the process of stopping point extractions from GPS trajectories. In contrast to the DPC method which operates on all data, this algorithm adopts a trajectory-based processing approach, reducing the algorithm complexity to $O(n^{2})$ depending on the maximum trajectory length. Compared with traditional one-step methods addressing one trajectory at a time, the algorithm may take longer to run due to the use of two clustering steps, but there is no increase in time complexity. In addition, the algorithm has demonstrated improved accuracy in stopping point extraction compared to traditional one-step methods, making it a viable option for trajectory analysis. Therefore, with each step having a clear physical meaning, the proposed two-step clustering algorithm can effectively extract stopping points based on latitude, longitude, and time information from GPS data. In the future, we will investigate the optimization of parameters and explore the combination of other classifiers to enhance the accuracy of stopping point extraction. 

## Figures and Tables

**Table 1:** Data format of trajectory.

Latitude	Longitude	Filed3	Altitude	Date (Number of Days)	Date	Time
39.984094	116.319236	0	492	39,744.2451967593	23-10-2008	05:53:05
39.984198	116.319322	0	492	39,744.2452083333	23-10-2008	05:53:06
39.984224	116.319402	0	492	39,744.2452662037	23-10-2008	05:53:11
⋯	⋯	⋯	⋯	⋯	⋯	⋯
39.977897	116.326624	0	310	39,797.0216203704	15-12-2008	00:31:08
39.977882	116.326626	0	310	39,797.0216782407	15-12-2008	00:31:13
39.977879	116.326628	0	310	39,797.0217361111	15-12-2008	00:31:18

**Table 2:** Comparison of different algorithms.

	DBSCAN-TE	One-Step DPCC-TE	Two-Step DPCC-TE
Accuracy	91.55%	92.12%	95.49%
Precision	68.57%	70.07%	85.02%
Recall	100.00%	100.00%	91.67%
F1 Score	0.8136	0.8240	0.8822
Time-consuming	7 m 6.7 s	7 m 29.6 s	11 m 44.9 s

## Data Availability

The data presented in this study are available upon request from the corresponding author. The data are not publicly available due to ethics.
